# CryoEM structure and small-angle X-ray scattering analyses of porcine retinol-binding protein 3

**DOI:** 10.1098/rsob.240180

**Published:** 2025-01-22

**Authors:** Vineeta Kaushik, Luca Gessa, Nelam Kumar, Matyáš Pinkas, Mariusz Czarnocki-Cieciura, Krzysztof Palczewski, Jiří Nováček, Humberto Fernandes

**Affiliations:** ^1^ Institute of Physical Chemistry, Polish Academy of Sciences, Warsaw, Poland; ^2^ Integrated Structural Biology Group, International Centre for Translational Eye Research, Institute of Physical Chemistry, Polish Academy of Sciences, Warsaw, Poland; ^3^ CEITEC Masaryk University, Kamenice 5, Brno 62500, Czech Republic; ^4^ Laboratory of Protein Structure, International Institute of Molecular and Cell Biology, Warsaw, Poland; ^5^ Departments of Ophthalmology, Chemistry, Physiology & Biophysics, and Molecular Biology & Biochemistry, Gavin Herbert Eye Institute-Center for Translational Vision Research, University of California, Irvine, CA 92697, USA

**Keywords:** RBP3, cryoEM structure, SAXS, conformational changes, molecular docking

## Abstract

The vertebrate visual cycle hinges on enzymatically converting all-*trans*-retinol (at-ROL) into 11-*cis*-retinal (11c-RAL), the chromophore that binds to opsins in photoreceptors, forming light-responsive pigments. When struck by a photon, these pigments activate the phototransduction pathway and initiate the process of vision. The enzymatic isomerization of at-ROL, crucial for restoring the visual pigments and preparing them to receive new light stimuli, relies on various enzymes found in both the photoreceptors and retinal pigment epithelium cells. To function effectively, retinoids must shuttle between these two cell types. Retinol-binding protein 3 (RBP3), located in the interphotoreceptor matrix, probably plays a pivotal role in this transport mechanism. Comprised of four retinoid-binding modules, RBP3 also binds fatty acids, potentially aiding retinal function by facilitating the loading and unloading of different retinoids at specific cell types thereby directing the cycle. In this study, we present a 3.67 Å cryoEM structure of porcine RBP3, along with molecular docking analysis and corroborative in-solution small-angle X-ray scattering data for titration of RBP3 with relevant ligands, that also give insights on RBP3 conformational adaptability.

**Figure:**
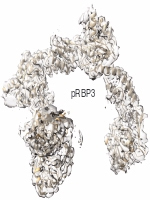

## 1. Introduction

The signal transduction of the vision process occurring in the photoreceptors depends on insoluble lipidic molecules known as retinoids [1]. All-*trans*-retinol (At-ROL) is delivered through the blood circulation to retinal pigment epithelium (RPE) cells, and after metabolic conversions and enzymatic isomerization of at-ROL, the resultant 11-*cis*-retinal (11c-RAL) can cross the interphotoreceptor matrix (IPM), entering the photoreceptors and binding to the opsins to form the light-responsive visual pigments. After photoisomerization of the pigments and enzymatic reduction of the hydrolytically released at-RAL, the at-ROL needs to be shuttled to the RPE for further processing to complete the cycle [2].

The IPM serves the function of transfer of retinal to the RPE and regulates not only the distribution of retinoids but also transport of oxygen and nutrients [3]. The shuttling of insoluble retinoids across the IPM involves the retinol-binding protein 3 (RBP3), which is a large approximately 140 kDa glycoprotein, present in almost all vertebrates. RBP3 resides mainly in the IPM [4], and it transports retinoids, fatty acids such as docosahexaenoic acid (DHA), and other hydrophobic molecules like retinoic acid, cholesterol and alpha-tocopherol [5], between the RPE and the photoreceptors, playing an important role in the visual cycle [3,6]. However, the molecular mechanisms of loading and unloading of RBP3 and export of retinoids across the IPM are not known.

The localization of RBP3 in the IPM is altered by light/dark conditions [7]. In light conditions, RBP3 is associated with the membranes of the RPE and photoreceptors [8,9], whereas in dark conditions, it appears to be more homogenously distributed across the IPM [7]. The quantities of RBP3 recovered from light- and dark-adapted eyes appear to be independent of the state of adaptation [10]. The binding of retinoids to RBP3 protects the photoreceptors by preventing photodegradation of the retinoids, and it regulates their transport across the IPM [11].

Expression of RBP3 in developing mice occurs prior to establishment of the visual cycle [12–14], and its cysteine residues may play a role in maintaining the redox balance of the healthy retina [6,15]. Malfunctions of RBP3 are believed to contribute to the pathologies of several retinal diseases [6], including diabetic retinopathy (DR) [16–18], retinitis pigmentosa (RP) [19,20], pan-retinal degeneration [21] and exaggerated eye growth and myopia [22–25].

Based on molecular characteristics, RBP3 consists of four tandem homologous modules of approximately 300 amino acids each, but elucidation of the three-dimensional structure of the intact multi-modular RBP3 has been largely evasive. Two crystal structures are available for single modules of the RBP3 protein; namely, RBP3 Module 1 from *Danio rerio* in complex with oleic acid [26] and RBP3 Module 2 from *Xenopus laevis* [27]. These structures of the isolated modules provide details of the protein’s molecular fold. Homology modelling and molecular docking results from these studies suggest that at least one of the ligand binding sites is located in the hydrophobic groove between domains A and B of the modules [28]. While this cleft may represent the retinoid-binding site, other studies suggest that the hydrophobic cavity within domain B is the major retinoid-binding site [26,29]. A lower resolution Cryo-electron microscopy (cryoEM) structure has been reported for full-length bovine RBP3 (bRBP3; PDB ID: 7JTI) in a complex with a antigen-binding fragment (FAB) [30].

In the current work, we have determined the structure of the RBP3 protein from the porcine (*Sus scofa*; pRBP3) retina at a higher resolution, using cryoEM. Besides the atomic structure, this work shows how retinoid binding changes the conformation of the four-module protein. Previous studies have suggested that binding of retinoids and fatty acids could impact the conformation of RBP3 and potentially modulate the interaction of RBP3 with other proteins and thereby regulate its overall function within the visual cycle. In the current study, we focused on this conformational adaptability, examining the RBP3 protein in solution with the small angle X-ray scattering (SAXS) technique and molecular docking.

## 2. Material and methods

The following compounds were all obtained from Sigma: at-ROL, oleic acid, retinoic acid and DHA. 11c-RAL was obtained from the National Eye Institute, National Institutes of Health, USA.

### 2.1. Isolation of IPM proteins

Fresh porcine (*Sus scrofa*) eyes were bought from a local slaughterhouse and kept on ice, in the dark. The eyes were dissected under dim red light, and the excised retinas were put on ice for immediate use, or frozen in liquid nitrogen and stored at −80℃. A volume of around 100 ml of excised retinas was gently stirred for 1 h at 4℃ in a 1:1 ratio with Buffer A (50 mM 4-(2-hydroxyethyl)-1-piperazine ethanesulfonic acid (HEPES) HCl, pH 8.0, 300 mM NaCl, 0.1 mM phenylmethylsulfonyl fluoride and 1 mM dithiothreitol (DTT)). IPM supernatant was separated from the retinas by centrifugation at 10 000g for 25 min at 4℃; Buffer B (1 mM CaCl_2_, 1 mM MgCl_2_ and 1 mM MnCl_2_) was added to the supernatant, and the mixture was poured over a glass wool-lined funnel and centrifuged at 20 000g for 90 min at 4℃, then the supernatant was filtered through a 0.45 µm syringe filter to obtain a preparation of IPM proteins.

### 2.2. Purification of RBP3

Concanavalin A (ConA)-Sepharose 4B (Cytiva) resin was used for isolating glycoproteins from the IPM-protein solution. The ConA-sepharose was equilibrated with Buffer B. After equilibration, the previously filtered supernatant was loaded on the resin and left on the rocker at 4℃ for 3 h. Using a gravity column, the resin was washed with 100 ml of Buffer B at 4℃ and eluted with 50 ml of MDM buffer (50 mM HEPES HCl, pH 8.0, 0.1 M NaCl and 400 mM methyl α-D-mannopyranoside). The purest fractions were pooled, and then desalted by ultra-filtration and re-dilution with Buffer C (50 mM HEPES HCl, pH 8.0 and 1.0 mM DTT), using a 100 kDa cut-off Amicon centrifugal filter.

The intermediate purification step was performed with anion exchange chromatography (AEC). The Amicon retentate was loaded onto a HiTrap Q HP 1 ml column (Cytiva). The column was then washed with 10× column volume of Buffer C. The elution was performed with a salt linear gradient with 10× column volume up to 1 M NaCl in Buffer C. The purest fractions were combined, concentrated to 500 µl and centrifuged at 20 000g at 4℃ for 1 h to remove precipitates.

The sample obtained from the AEC step was then loaded onto a Superdex 200 Increase 10/300 GL column (Cytiva) for gel filtration chromatography and eluted with 50 mM HEPES HCl, pH 8.0, 300 mM NaCl, 1 mM DTT and 0.01% n-dodecyl-β-D-maltoside. All fractions showing absorbance at 280 nm were analysed by sodium dodecyl sulfate-polyacrylamide gel electrophoresis (SDS-PAGE), showing a very high degree of purity.

For obtaining deglycosylated RBP3 after anion-exchange purification, the protein was incubated overnight with PNGaseF enzyme, and the glycan moieties and PNGaseF were removed by size exclusion chromatography (SEC), as described above.

### 2.3. Fluorescence retinoid binding assay

To check that RBP3 retained its functionality, its ability to bind at-ROL, 11c-RAL, retinoic acid, oleic acid and DHA was confirmed by a fluorescence assay, using a 4.4 μM solution of RBP3 maintained in dark conditions. The assay was performed using a Perkin Elmer instrument (Varian Cary Eclipse Fluorescence Spectrophotometer). Titrations (0−20.0 µM, at-ROL, 11c-RAL, all-*trans* retinoic acid or fatty acids) were performed at room temperature in 50 mM HEPES HCl buffer, pH 8.0, containing 300 mM NaCl, 0.1 mM DTT and 5% glycerol (v/v). The retinoid molecules (in a crystalline form) were dissolved initially in ethanol, then diluted into the HEPES buffer. The ethanol content in the final sample solutions was kept below 2% by volume.

### 2.4. CryoEM and data collection

Purified *S. scofa* RBP3 protein (pRBP3), concentrated to 4 mg ml^−1^ at 4℃, was diluted prior to grid preparation. To obtain the cryoEM structure of apo pRBP3, 3 µl of a solution of RBP3 at a concentration of 0.8 mg ml^−1^, and containing 0.5% 3-(3-Cholamidopropyl]dimethylammonio)-2-hydroxy-1-propanesulfonate (CHAPSO), was deposited on a plasma-cleaned Quantifoil R2/1 200 mesh (EM sciences). For the complexes with retinoids and fatty acids, pRBP3 at 0.9 mg ml^−1^ protein concentration along with 0.5% CHAPSO was incubated with different concentrations (2, 8 and 12 µM) of retinoids (at-ROL and 11c-RAL) and DHA; and 3 µl was deposited on the grids. Protein excess was blotted for 4 s with 0 blot force at 4℃ under 95% humidity before plunge-freezing the sample in liquid ethane, using the Vitrobot Mark IV (ThermoFisher) system. A Talos Arctica (Thermo Fisher) cryoEM instrument was used, operating at 200 kV and equipped with a K2 Summit direct electron detector and Bioquantum Imaging filter. The best grid was used for data collection on a Titan Krios G1 microscope (ThermoScientific) operated at 300 kV, aligned for fringe-free imaging and equipped with a K3 direct electron detector and BioQuantum Imaging Filter (Ametek). The data were collected in electron-counting mode at 105 000× magnification, corresponding to the pixel size of 0.8336 Å, with the energy slit set to 10 eV. The data collection was performed using SerialEM software [31]. A total of 23 971 movies were collected in a dose-fraction mode, with each movie consisting of 40 frames with a dose of 1 e Å^−2^ in each; and a total dose of 40 e Å^−2^ over a 2 s exposition.

### 2.5. Image processing and model building

CryoEM data were processed with the cryoSPARC package (Structura Biotechnology) [32]. Briefly, motion correction was performed by full-frame motion correction, and subsequent micrographs were contrast transfer function (CTF)-corrected using CTFFIND4 [33]. Micrographs were curated to include CTF fits better than 4.5 Å and astigmatism less than 400 Å. A fraction of the micrographs were used for blob picking to locate and then extract some particles, followed by repetitive runs of two-dimensional classification that generated 176 000 particles, which were then used for low-resolution initial structure and two-dimensional templates. More than 7 462 000 potential particle candidates were then identified by Template picker and were further analysed by multiple two-dimensional classification runs; 1 903 572 particles that displayed clear secondary structural features were selected and used for downstream processing. Selected particles were used in a repetitive hetero-refinement run with multiple initial structures as the volume input, effectively three-dimensional—classifying the dataset and narrowing the number of good particles down in every step. After each hetero-refinement step, local refinement was performed to reconstruct a Coulomb potential map, with the best map being obtained from 611 246 particles after three cycles. The map sharpened with one b factor (−179.4 Å^2^) was resolved at 3.67 Å resolution (according to the Fourier Shell Correlation (FSC) = 0.143 criterion), with both flexible arms of the structure being smeared. To provide a more meaningful consensus map, these arms were then reconstructed using the three-dimensional-flex algorithm.

The atomic model based on pRBP3 AlphaFold prediction (entry AF-A0A287A908-F1) was docked onto the resulting apo pRBP3 cryoEM map. Several rounds of iterative cycles of manual model building, using COOT [34]; and real space refinement, using PHENIX [35] were carried out to get the final model of apo pRBP3. The quality of the structure was monitored using MolProbity [36].

### 2.6. Molecular docking

The rigid-core pRBP3 structure (from E417 to P1191) was selected as the receptor, and 11c-RAL, at-ROL, DHA and oleic acid were used as ligands. Docking simulations were performed with CB-Dock2 [37] on the prepared libraries and searched for 20 cavities/ligands. CB-Dock2 is an advanced protein–ligand docking technique whose ability to autonomously identify potential ligand-binding sites is complemented with an advanced AutoDock Vina prediction of the exact binding position of the queried ligand.

### 2.7. Small angle X-ray scattering

SAXS data were collected at the P12 undulator beamline at the Petra III storage ring at DESY (Hamburg, Germany). SAXS measurements were carried out for apo pRBP3 at 0.3 mg ml^−1^ (2.22 µM in 50 mM HEPES HCl buffer, pH 8.0, containing 300 mM NaCl, 0.1 mM DTT); subsequently the apo RBP3 was titrated with different retinoids and fatty acids (e.g. at-ROL, 11c-RAL, oleic acid and DHA) with stepwise increases in the concentration of each compound from 0 to 20 µM. The complexes were formed by rapid mixing (on the order of a few seconds), by pippetting up and down, and immediately scanned on the beamline. The compounds were delivered in ethanol, whose concentration never exceeded 2% v/v of the total sample. Data collection was performed at 20℃, and 40 frames of 1 s were collected for each sample (electronic supplementary material, table S1–S4). Buffer scattering was measured to generate buffer-subtracted intensity profiles, and the data were processed using Primusqt implemented in ATSAS 2.8.1 [38]. The forward scattering I(0) and radius for each complex were estimated using AUTOGNOM, which was further used to evaluate the molecular size by plotting a pair-distance distribution function. P(R) of scattering data is the representation in real space and reflects the particular shape. P(R) approaches zero at its maximum dimension *D*_max_. AUTOGNOM results were used to generate 10 independent *ab initio* models through DAMMIF and Gasbor [38].

## 3. Results

### 3.1. RBP3 purification

**Figure 1.**
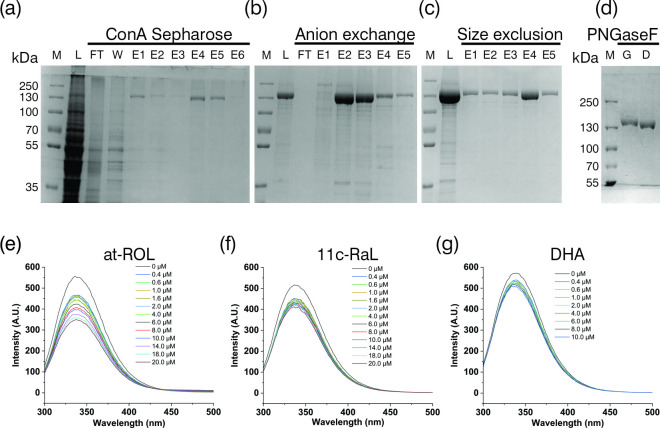
Purification and biochemical characterization of purified porcine RBP3. SDS-PAGE analyses are shown samples from the three consecutive chromatographic steps (*a–c*), and of the PNGase-F-deglycosylation step (*d*). (*e*) Representative fluorescence spectra of pRBP3 upon titration with at-ROL. (*f*) Representative fluorescence spectra of pRBP3 upon titration with 11c-RAL. (*g*) Representative fluorescence spectra of pRBP3 upon titration with DHA.

Retinas were isolated from porcine eyes, and the corresponding RBP3 protein was purified using ConA chromatography followed by anion exchange and SEC techniques (figure 1*a*–c). In addition, to prevent aggregation, DTT and high salt concentration were used throughout the purification process. Detergent concentration was maintained below the micelle-formation threshold during the SEC. The purified proteins were further used for biophysical and structural characterization. For deglycosylation, the PNGaseF enzyme was used, and the impact of deglycosylation on the electrophoretic mobility of the purified protein was assessed using SDS-PAGE. A noticeable size difference between the glycosylated and deglycosylated RBP3 proteins was observed (figure 1*d*).

RBP3 elutes from SEC as a monomer; and purification in the absence of a reducing agent (DTT) also yielded monomeric RBP3, but it was more prone to aggregation, judged by the presence of higher molecular weight SEC peaks, difficulty in achieving a concentrated solution, and visible aggregates on TEM images of cryoEM grids (data not shown).

### 3.2. Intrinsic fluorescence of RBP3 protein with retinoids

The activity of the purified RBP3 was assessed by observing changes in fluorescence resulting from titration of the protein with ligands. During the titration with retinoids, two maxima were observed at *λ* 338 and 480 nm in the emission spectrum, corresponding to Trp residues of the RBP3s and to at-ROL, respectively (electronic supplementary material, figure S1 ). Porcine RBP3 displayed the expected preference for 11c-RAL over at-ROL when isolated from dark-adapted retinas (figure 1*e*,*f*). Our measurements indicate a stronger binding to retinoids than fatty acids (figure 1*e*,*f*,*g*). The absence of a reducing agent did not change RBP3 binding capacity (data not shown).

### 3.3. pRBP3 cryoEM structure

The first full-length RBP3 structure was determined in 2020, assisted by complex formation with a monoclonal antibody that bound to Module 2 and resulted in a 7.4 Å-resolution reconstruction [30]. The antibody formed a non-native complex with the RBP3 protein. To obtain an intact RBP3 structure, we used pRBP3 in the presence of CHAPSO, without antibodies or fab fragments (when tested they were detrimental to obtaining good-quality particles).

Screening the grids with apo pRBP3 and the three liganded complexes revealed proper particle distribution (table 1), which could be allocated to distinct two-dimensional classes (electronic supplementary material, figure S2). Of note, neither the apo protein nor the complexes showed any significant presence of elongated particles, as reported by Sears *et al.* [30]. Of the four datasets, the one with apoprotein-generated data could be processed to higher resolution, so it was the only one processed further and analysed (table 1).

**Table 1.** CryoEM data collection parameters and refinement and validation statistics. n.a. = not applicable.

	native pRBP3	pRBP3 +11c-RAL	pRBP3 +at-ROL	pRBP3 +DHA
data collection processing				
magnification	105 000×	165 000×	165 000×	165 000×
voltage (kV)	300	200	200	200
electron exposure (e Å^−2^)	40	40	40	40
defocus range (μm)	−1.4 to −3.0	−1.6 to −3.0	−1.6 to −3.0	−1.6 to −3.0
physical pixel size (Å)	0.8336	0.783	0.783	0.783
symmetry imposed	C1	C1	C1	C1
initial particle images (*n*)	1 903 572	18 582	15 647	18 027
final particle images (*n*)	611 246	n.a.	n.a.	n.a.
map resolution (Å)	3.67			
FSC threshold	0.143			
map resolution range (Å)	3.2 to 7.0^a^			
refinement				
model resolution (Å)	4.42			
FSC threshold	0.5			
model composition	Apo pRBP3			
non-hydrogen atoms	8324			
protein residues	1081			
ligands	0			
B factors (Å^2^)				
protein	208.6 (Rigid = 75.1) (Flexible = 363.2)			
ligand	n.a.			
Rms deviations				
bond lengths (Å)	0.003			
bond angles (°)	0.546			
validation				
MolProbity score	2.56			
clash score	9.88			
poor rotamers (%)	0.21			
Ramachandran plot				
favoured (%)	96.01			
allowed (%)	3.99			
disallowed (%)	0			

^a^ The resolution for the flexible parts, due to their intrinsic flexibility, is probably overestimated, and some random correlation of high-frequency information is unintentionally obtained.

**Figure 2.**
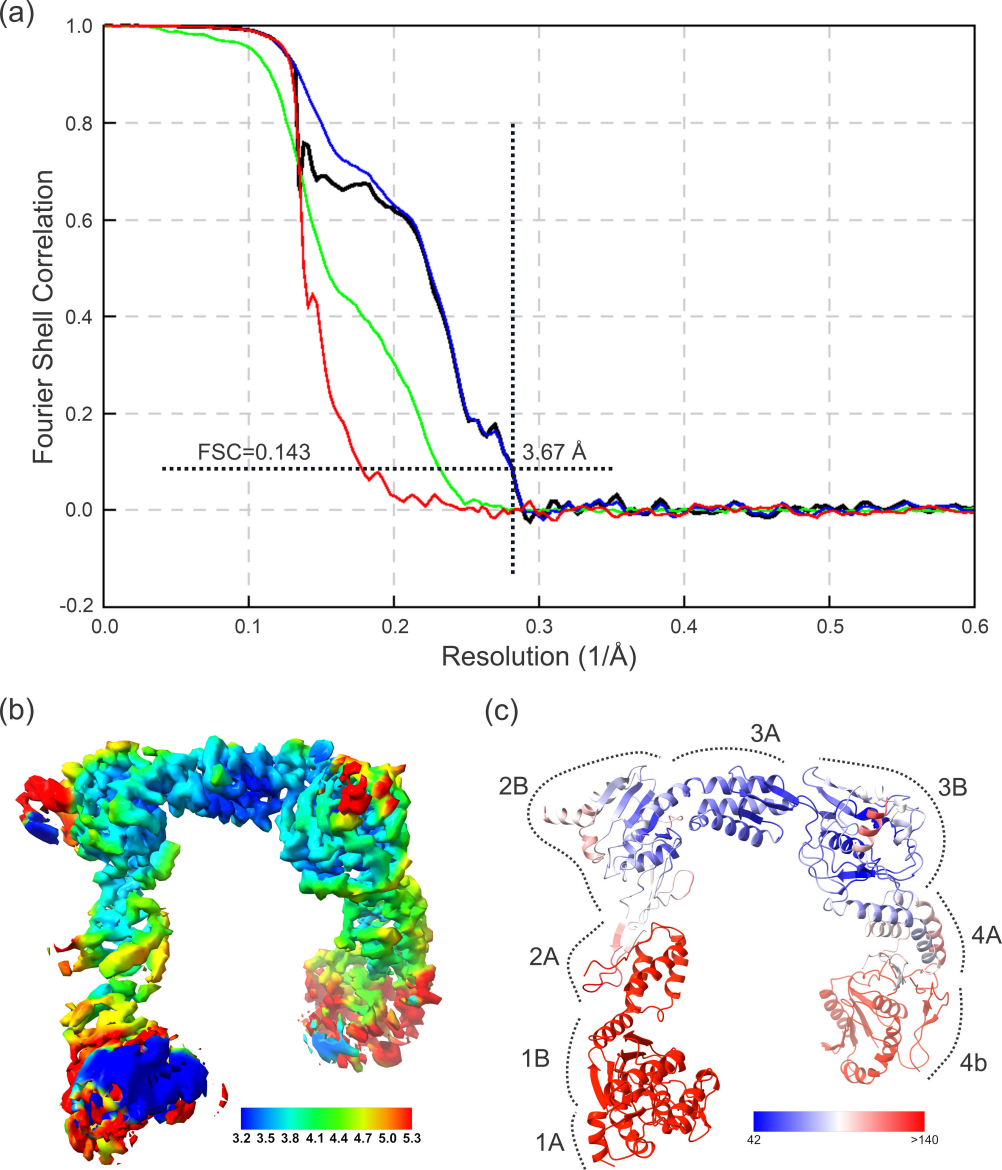
Global resolution, local resolution map and refined pRBP3 structure. (*a*) Curves of FSC computed for phase-randomized masked half-maps (red), unmasked half-maps (green), masked half-maps (blue) and the corrected FSC curve (black). (*b*) CryoEM-density map filtered and coloured according to local resolution. (*c*) Cartoon representation of the refined atomic model of pRBP3 coloured by residue-averaged atomic B factor.

**Figure 3.**
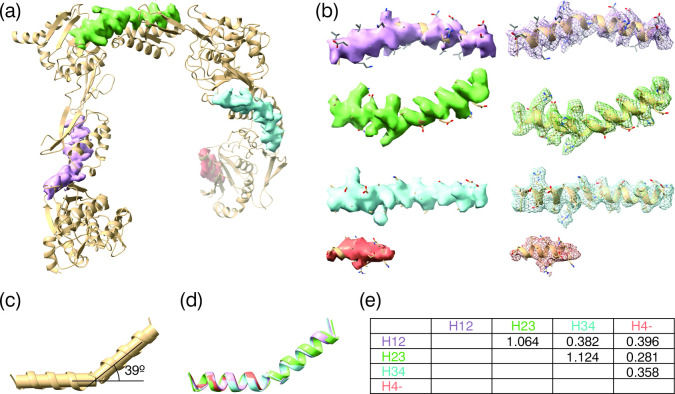
Modules-connecting helices. (*a*) Cartoon representation of the refined atomic model of pRBP3 with the cryoEM map displayed for just the modules-connecting helices, with surface representation of the density. (*b*) Enlarged representation of the individual helices, and additional a mesh representation of the density. (*c*) Angle generated by the kink in the helix (H12) joining Modules 1 and 2. (*d*) Overlap of the four helices to highlight the small rmsd and conserved kink position. (*e*) Rmsd in Å of the four helices that connect the four pRBP3 modules.

Hetero-refinement, followed by local refinement provided a final resolution of 3.67 Å (figure 2*a*; electronic supplementary material, figure S3). The local resolution map analysis (figure 2*b*) revealed that the stretch from domain B of Module 2 to domain A of Module 4, inclusive, displays good parameters and could be refined de novo (accordingly, this region was used for the description of the structure analysis); the maps for the remaining portions of RBP3, at both termini, allowed only rigid body fit. Such a split is also clearly visible when the B factors are analysed, with lower values (75.1 Å^2^, average, for 583 residues) for the rigid part, and very high (363.2 Å^2^, average, for 498 residues) for the flexible ones (figure 2*c*). The map displays the four pRBP3 modules, which adopt a ‘pi’ shape fold, similar to the bovine variant [30], and have good density for the connected alpha helices that bridge the individual modules (figure 3*a*,*b*).

**Figure 4.**
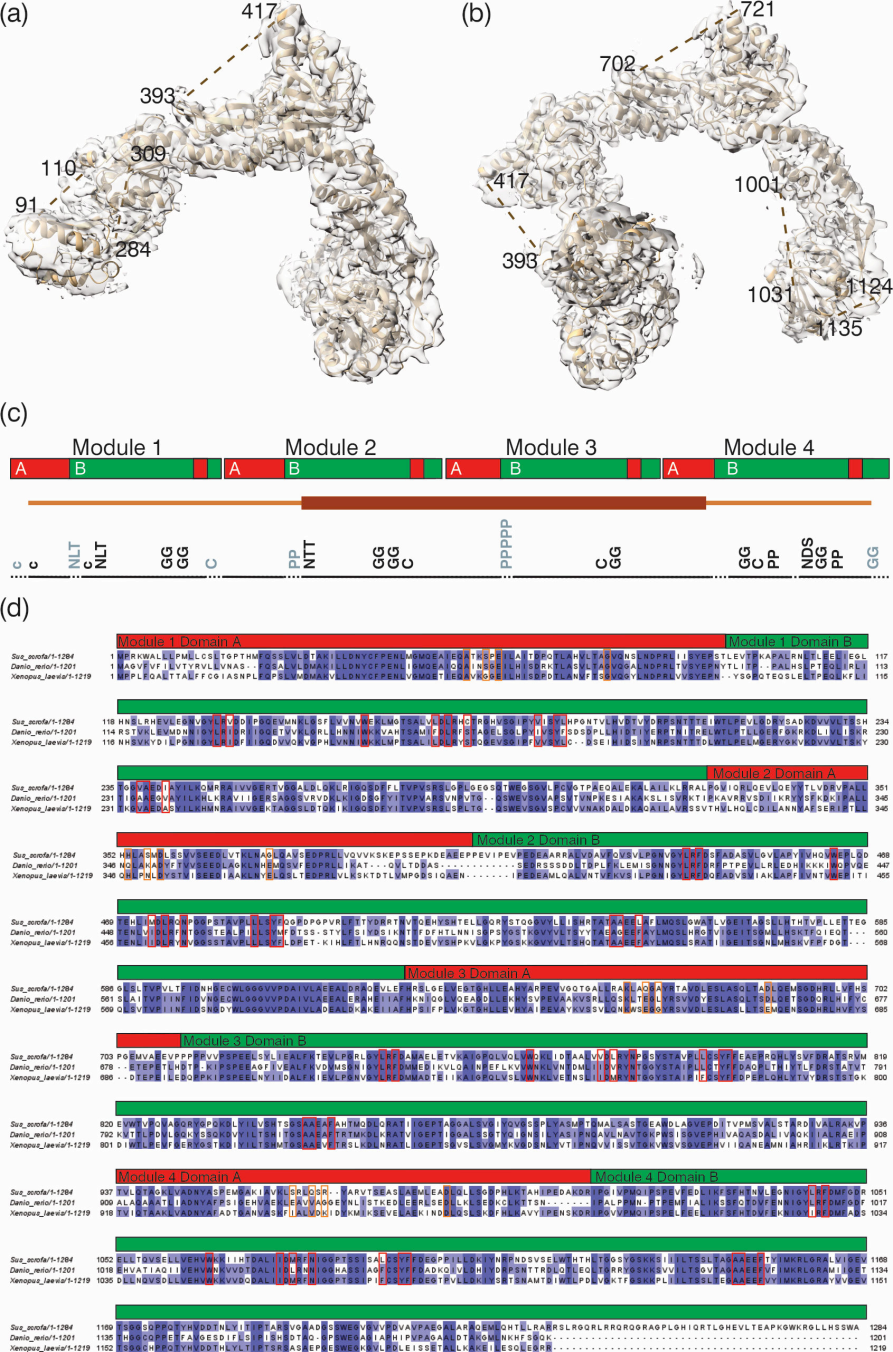
Missing loops, and module and domain organization of pRBP3. (*a–b*) pRBP3 loops that lack density. (*c*) Linear representation of the four pRBP3 modules, and split of domains A and B. Also shown are the rigid (dark-brown) and flexible (light-brown) distribution (middle line), the missing loops in the structure (dotted black line) and some relevant amino acid positioning (bottom black line). (*d*) Oleic acid binding sites in the context of sequence alignment of porcine RBP3 protein (NCBI Reference Sequence: XP_020928418.1), and its *Danio rerio* (NCBI Reference Sequence: XP_017213919.1) and *Xenopus laevis* (NCBI Reference Sequence: AAI70559.1) homologues, performed using the algorithm Clustal omega embedded in the software Jalview [39–41]. Residues identical in all sequences are shaded in dark blue, while those conserved in two of the homologues are shaded in lighter blue. The red boxes outline residues involved in oleic acid binding at the hydrophobic cavity within domain B, as determined by docking, and the orange boxes outline residues involved in oleic acid binding to domain A, as determined by X-ray crystallography (PDB id: 4LUR). The module and domain splits correspond to the porcine RBP3 structure.

The long module-connecting alpha-helices are bent after turn three, with an angle of approximately 39° (figure 3*c*) and overlap with a root mean square deviation (rmsd) of 0.38−1.12 Å (figure 3*d*,*e*). Each module consists of two domains that are bridged by a disordered linker, not visible on our density maps. The maps also lack density at the N- and C-termini and at additional connecting loops (figure 4).

**Figure 5.**
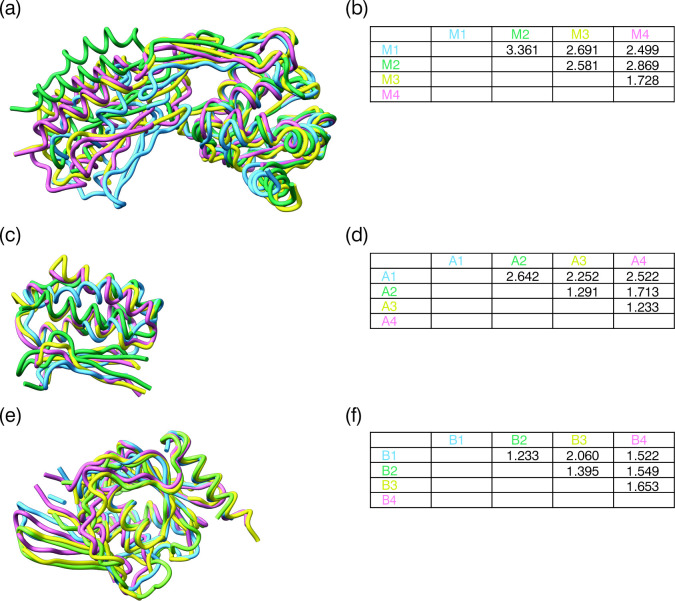
Superposition of pRBP3 modules and domains, and protein-topology structure. (*a,c,e*) superposition of the four pRBP3 modules, domain A and domain B, respectively. (*b,d,f*) Rmsd calculation for all pairs of combinations for the four modules, domain A and domain B, respectively.

The four modules overlap well with a rmsd between 1.73 and 3.36 Å (figure 5*a*,*b*). Domain A is formed by a three-helix bundle followed by a small β strand (using the first 80 residues of the primary sequence) that forms a three-strand anti-parallel β-sheet by the contribution of a two-strand insertion from 20 residues in the middle of domain B (figures 4*c* and 5*c,d*). This domain is responsible for binding fatty acids [26]; but on our maps (due to the resolution and/or the ligands being washed out during purification [42]), no density is visible at the binding sites. Domain B starts with a six-stranded large mixed β-sheet with four helices packing on one side and the fifth and last helix packing on the other side. A second sheet is then formed by four antiparallel β-strands that extend toward domain A and form an open, mostly hydrophobic, cleft between the two domains (figure 5*e*,*f*). The two domains come together with the extension of the fifth strand of the larger sheet and the third strand of the small sheet of domain B, together with the small β strand of domain A, packing together and against the N-terminal three-helix bundle of domain A (figures 4*c* and 5*a*).

The amino acid sequence of porcine RBP3 shows four instances of at least two consecutive prolines, which may influence the tertiary structure; in Modules 3 and 4 these prolines are located on the loop that connects domains A and B, with the stretch in Module 3 being of five prolines, but its relevance for the structural fold of the modules cannot be discerned due to the lack of density (figure 4*c*). The pRBP3 primary sequence also harbours seven cysteine residues, five of which could be modelled and do not engage in disulfide bonds (figure 4*c*). The remaining two cysteines are located at the disordered N-terminus (C14) and on a disordered loop within domain B of Module 1 (C304), and they would be too far apart from other cysteines to engage in intraprotomer disulfide bonds. Therefore, it is likely that monomeric porcine RBP3 has seven free thiols. pRBP3 is also predicted to harbour four N-glycosylation sites (figure 4*c*), all with well-defined densities for the protein polypeptide attaching point, but no traces of the glycan moieties are found on our cryoEM map.

Structure homology searches with DALI [43] identify several proteases with the highest scores for a putative peptidase from *Parabacteroides merdae* ATCC 43184 (PDB id: 4L8K), the secreted chlamydia protease CPAF (PDB id: 3DJA), a putative S41 protease (YP_211611.1) from *Bacteroides fragilis* NCTC 9343 (PDB id: 3K50), and tail-specific protease (PDB id: 6IQR).

### 3.4. pRBP3 structural features—implication in disease

RP is associated with a D1080N mutation, on a residue that is located in domain B of Module 4 of RBP3. Inspection of the structure suggests that the mutation would result in a shift of hydrogen bonding. The Asp 1080 hydrogen bond with Arg 1082 in the wild-type (WT) protein may be replaced with a hydrogen bond between the new Asn 1080 with the neighbouring Tyr 1044. Such changes alter the protein surface and thus may impact the interactions of RBP3 with endoplasmic reticulum chaperones, and in turn trap RBP3-D1080N in the ER [19].

### 3.5. pRBP3 putative binding sites—docking

To search for potential binding sites for retinoids and fatty acids in the mammalian RBP3 modules, we used docking methodology and tested pRBP3 complexes with 11c-RAL, at-ROL, DHA and oleic acid (OA). We decided to search for 20 binding sites for each ligand, using a number of potential sites well above what is expected physiologically, with the prospect that the relative binding energies would distinguish the stronger binding sites from the weaker ones (electronic supplementary material, table S1, figure S4). Of note, the binding site identified on the Zebrafish module co-crystallized with oleic acid [26] was not identified with our screens of the conformation adopted by pRBP3 in our cryoEM structure (figure 4*d*) despite relatively high sequence and structure similarities (table 2).

**Table 2.** Comparison of *Sus scrofa* RBP3 with other organisms’ RBP3 with structures determined by X-ray crystallography.

comparison	rmsd (Å)	sequence identity (%)	sequence similarity (%)
*Sus scrofa* versus *Xenopus laevis* RBP3	1.137	58	76
*Sus scrofa* versus *Danio rerio* (Zebrafish) RBP3	1.039	50	70

rmsd: the root mean square deviation is used to compare the three-dimensional structural differences between the points.

Sequence identity (%) = percentage of identical residues in the aligned sequence.

Sequence similarity (%) = percentage of similar residues that have similar physiochemical properties.

**Figure 6.**
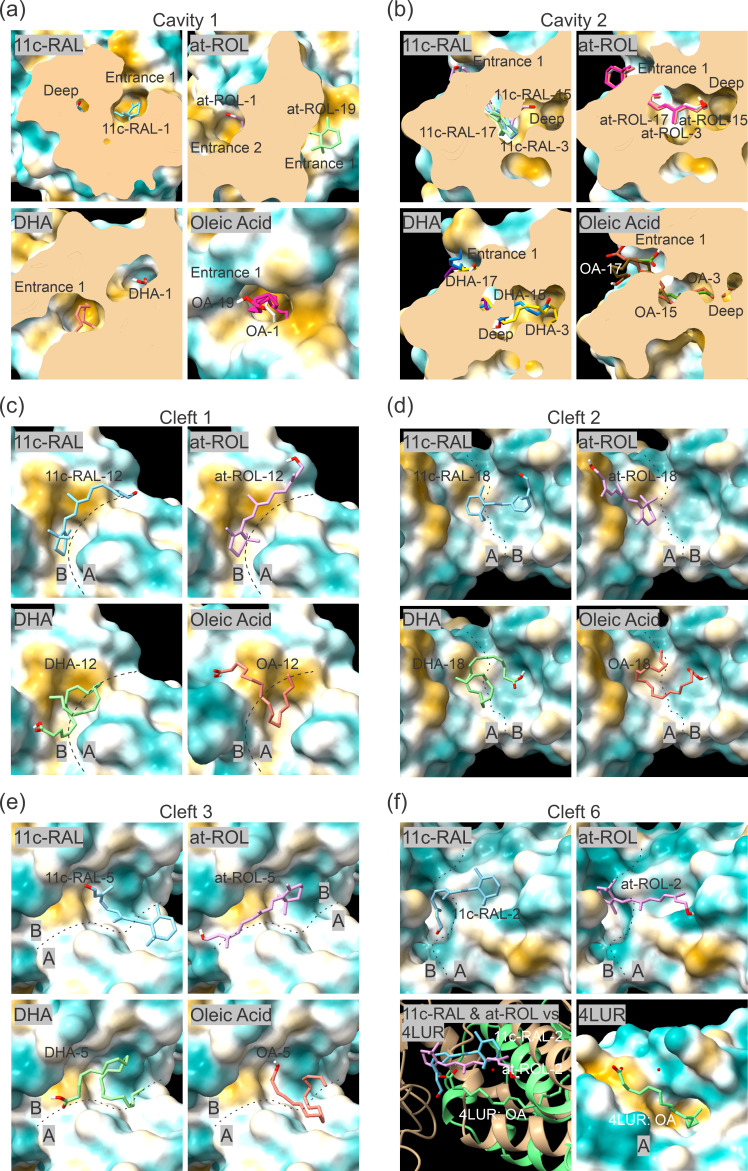
Molecular docking of 11c-RAL, at-ROL, DHA and OA in pRBP3. (*a–b*) Cavities 1 and 2 in domain B of pRBP3. (*c–f*) Clefts 1, 2, 3 and 6 occur at the interfaces of the pRBP3 domains (panel (*f*) also displays the aligned 4LUR structure and its OA ligand). The surface representation of pRBP3 is coloured according to relative hydrophobicity (golden colour the most lipophilic and dark cyan the most hydrophilic).

Our screen, using the rigid component of the pRBP3 structure, identified a deep binding cavity within domain B of Module 3 (Cavity 1) and Module 2 (Cavity 2), along with 18 clefts. The stronger binding is observed for the Cavity 1 (electronic supplementary material, table S1), with binding energies of −9.8, −8.6, −9.0 and −7.5 kcal mol^−1^ for 11c-RAL-1, at-ROL-1, DHA-1 and OA-1, respectively; 11c-RAL and DHA were fully inside the cavity, whereas at-ROL and OA were not as deep, and the orientation of the molecules suggested a second possible binding mode. Thus, at-ROL-1 has its polar moiety pointing to one cavity entrance, and at-ROL-19 has the β-ionone ring placed at another entrance, with its polar group inside the cavity and with a binding energy of −6.2 kcal mol^−1^. OA-19 is placed less deep than OA-1 with a binding energy of −5.4 kcal mol^−1^ (figure 6*a*). The volume of Cavity 2 is smaller than Cavity 1 (1807 versus 1939 Å^3^), but CB-Dock-2 still finds it possible for Cavity 2 to accommodate all of the tested ligands in three possible conformations/positions (figure 6*b*). The binding energies for Cavity 2 are weaker than for Cavity 1, but follow the same trend, with 11c-RAL being a stronger binder and OA being weaker (electronic supplementary material, table S1). This preference for binding 11c-RAL over OA is generally true for the 18 hydrophobic clefts populating the protein surface (electronic supplementary material, table S1). Clefts 1 and 2 are located between domains A and B of Module 3 and accommodate one of the ligands each (figures 6*c*,*d*; electronic supplementary material, figure S4). Of the three clefts that are located at the interface between domain B of Module 3 and domain A of Module 4 (Clefts 3−5), only Cleft-3 accommodates every ligand tested (figure 6*e*; electronic supplementary material, figure S4). The similar interface between domain B of Module 2 and domain A of Module 3 has two clefts (Clefts 6 and 7) almost co-localizing (electronic supplementary material, figure S4). Cleft 6 is only occupied by 11-RAL-2 and at-ROL-2, with DHA and OA not being reported binders (figure 6*f*).

Considering clefts that are located solely in a single domain, domain B contains two clefts in Module 2 (Clefts 8 and 9), and three clefts in Module 3 (Clefts 10, 11 and 12); and domain A has one cleft each on Module 3 (Cleft 13) and Module 4 (Cleft 14) that bind 11c-RAL, the preferred ligand.

### 3.6. Small-angle X-ray scattering

**Figure 7.**
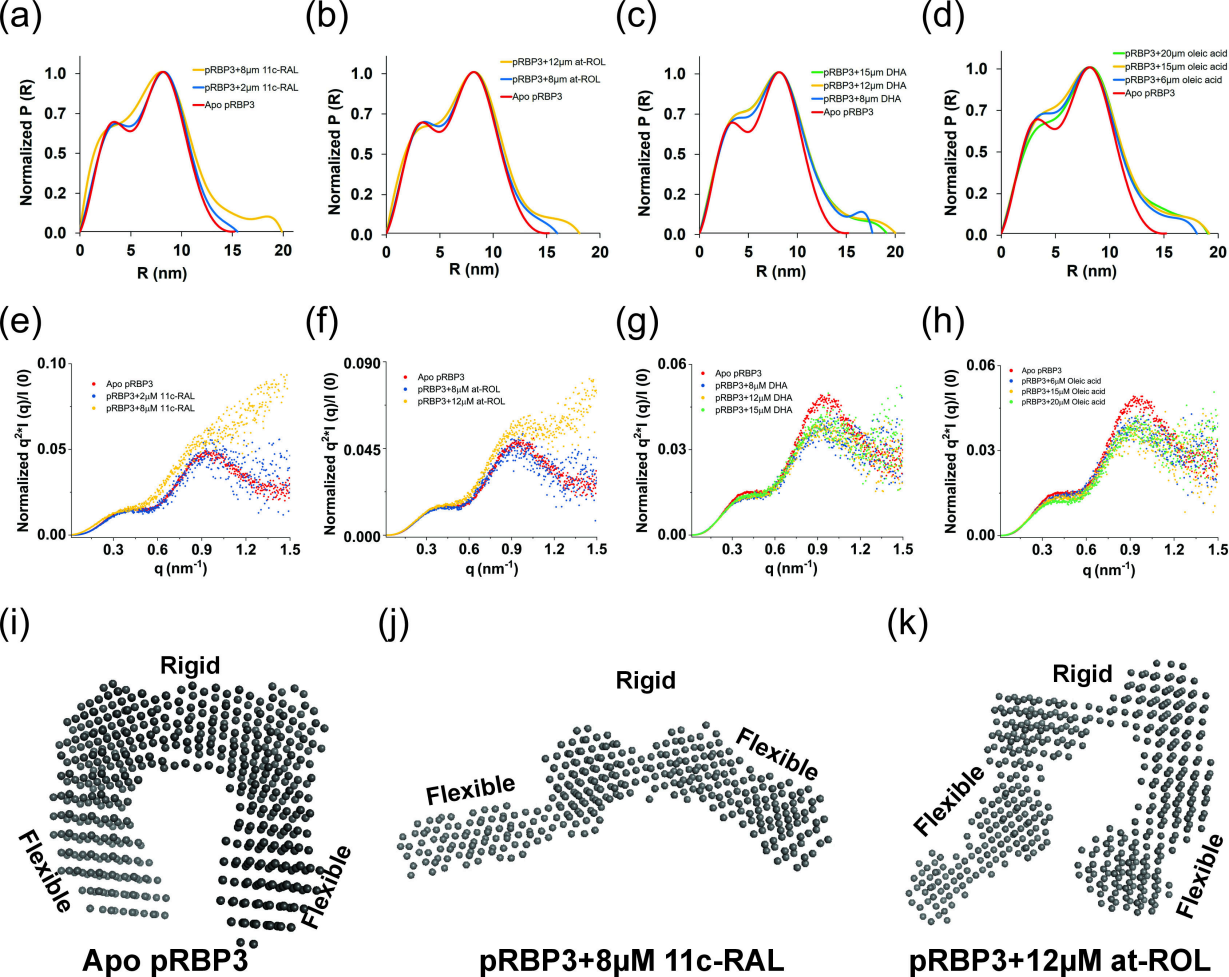
Structural dynamics of the pRBP3 protein alone and in complex with retinoids and fatty acids as determined by SAXS. (*a–d*) The normalized pairwise interatomic distance distribution P(R) function for apo pRBP3, and for the complexes of pRBP3 with retinoids or fatty acids demonstrate an increase in *D*_max_ value for the complexes. (*e–h*) Normalized Kratky plots of apo pRBP3 (red); and an increase in the degree of disorder of pRBP3 in complex with 8 μM 11c-ROL (a, yellow) or 12 μM at-ROL (b, yellow). No changes were observed in the presence of DHA (*g*) or oleic acid (*h*). (*i–k*) DAMMIF-averaged *ab initio* models of (*i*) apo pRBP3, (*j*) pRBP3 with 8 μM 11c-RAL and (*k*) pRBP3 with 12 μM at-ROL.

To investigate the discrepancy between the observation of an extended RBP3 molecule by Adler *et al.* [44] and the lack of such a conformation in our current study and that of Sears *et al.* [30], we performed SAXS on porcine RBP3, isolated from native sources (figure 7). The SAXS measurements for porcine RBP3 are in good agreement with the theoretical scattering curve calculated from the cryoEM structure, indicating that the pRBP3 monomer is stably assembled (figure 7). The experimentally determined radius of gyration (*R*_g_) for pRBP3 is 5.22 ± 0.73 nm, which matches with the atomic coordinates (PDB: 7JTI [30]). The P(R) distribution reflects a linear maximum dimension (*D*_max_) of 15.2 nm (figure 7*a*–*d*). The Kratky analysis demonstrates the globular nature and absence of a compact structure of the pRBP3; and Guinier analysis confirms the lack of aggregation in the protein sample (figure 7*e*–*h*; electronic supplementary material, figure S5). We studied the behaviour of the pRBP3-ligand complexes in solution at molar ratios up to 1:9 (protein:ligand). The calculated Rg values for the pRBP3−8 μM 11c-RAL complex was 5.94 ± 1.05 nm (electronic supplementary material, table S2); and for the pRBP3−12 μM at-ROL complex, 5.73 ± 1.03 nm (electronic supplementary material, table S3). Increases in the *D*_max_ values of the complexes (19.87 nm with 8 μM 11c-RAL, and 18.07 nm with 12 μM at-ROL) relative to the *D*_max_ for pRBP3 alone (15.2 nm; electronic supplementary material, table S2), suggested that ligand binding led to extension of the protein shape in solution. The Kratky plots for the pRBP3-8 μM 11c-RAL and pRBP3-12 μM at-ROL complexes plateau at higher *q* values, indicating flexibility (figure 7*e*,*f*). We could not observe any significant conformational changes with DHA or oleic acid, even though we observed an increased *D*_max_ for higher concentrations of these ligands (figure 7*a*–*d*).

We repeated the DAMMIF *ab initio* modelling for 10 iterations and generated averaged models for the samples. Concomitantly, our SAXS-generated bead models showed a ‘pi’-shape conformation for apo pRBP3 (figure 7*i*), and open ‘pi’-shape conformations in the presence of 11c-RAL (figure 7*j*) and at-ROL (figure 7*k*) in solution, although of limited extension compared with the case of bovine RBP3 [44]. The open (figure 7*j*) and partially opened (figure 7*k*) conformations of pRBP3 depict the protein having modules 2 and 3 unmoved, while Modules 1 and 4 were rotated in the three-dimensional environment compared with the apo conformation (figure 7*i*).

## 4. Discussion

Our understanding of the visual cycle and the relationship of RPB3 with retinal disease has been hampered by the lack of an experimental structural model of the native protein. Based on previous knowledge of RBP3 properties and straightforward methods for isolation of the porcine variant of RBP3 [45,46], we purified porcine RBP3, and obtained a protein with Förster resonance energy transfer (FRET) behaviour analogous to other RBP3s (figure 1*e*,*g*) [30]. Through analysis of cryoEM data, we determined a structure at 3.67 Å resolution of the porcine RBP3 protein and observed conformational changes upon ligand binding.

Structural homology of pRBP3 confirms previous findings of similarity between RBP3 and proteases whose structures utilize ββα-spiral folds to stabilize their ligand-binding sites [28,47–49]. Evolutionarily of bacterial origin, the specific IPM RBP3 variants have lost the protease catalytic residues after interdomain horizontal gene transfer into vertebrates [50], and instead exhibit retinoid and fatty-acid transport functions.

### 4.1. Structure/disease—RBP3 in pathology

The association of RBP3 with some pathologies seems to be related to the amounts of the RBP3 protein in the IPM (and/or vitreous) rather than to mutagenesis that would affect the protein function directly. For example, DR is linked to a decrease in RBP3 protein levels as is RP. A study conducted by Yokomizo *et al.* in 2019 suggests that RBP3 plays a major protective role in the progression to severe DR, identifying RBP3’s apparent ability to ameliorate the effects of hyperglycemia on retinal endothelial cells and Müller cells [17]. The molecular mechanism of the protective effect appears to be related to the binding of RBP3 antagonist to glucose transporter 1 receptor as well as to vascular endothelial growth factor and inhibiting tyrosine phosphorylation [17]. Currently, there is a significant need for biomarkers for the cure or the early detection of DR, and RBP3 could be a prototype in this context [51–53].

The decrease in RBP3 protein levels in RP is the consequence of a point mutation (D1080N) that traps the mutant RBP3 in the ER (impaired secretion), resulting in ER stress, with a gain of cytotoxic function [19]. Our structure shows that three residues may be involved in the pathology, with all three being conserved in the porcine sequence on the four modules; and this conservation is also observed in the human and bovine variants. The D1080N mutation may change the protein surface sufficiently to disrupt interactions with chaperones or other proteins located in the ER, preventing the exit of RBP3 [19]. Li *et al.* also reported that two cysteines (C304 and C1175), in the human variant, are responsible for formation of high molecular weight insoluble complexes [19]. Our structure shows that porcine S304 is on a disordered loop in domain B of Module 1, and C1175 on a loop between domain A and B of Module 4. We interpret our structure to indicate that the D1080N mutation does not contribute directly to the formation of abnormal disulfide bonds. Actually, high molecular complexes are present to a lesser extent than for the WT protein [19]. However, by interfering with RBP3 secretion the D1080N mutation may allow enough time and/or high local concentration to promote such disulfide bonding either with other RBP3 molecules or with other proteins.

Considering the association of RBP3 with myopia, the available data identify several mutations that suggest a loss of function may be the genetic mechanism of RBP3-related myopia, as it is for DR and RP. Nonsense mutations [24] that lead to truncated forms of the RBP3 protein definitely contribute to a loss of function. R170Q is located at the same patch (this time in Module 1) as the D1080N mutation, and perhaps its pathology may have to do with new hydrogen bonding or a new surface epitope that may lead to problems with ER progression. Likewise, the L540P mutation, which also does not seem to be involved in retinol binding, or other ligands (figure 4*d*; electronic supplementary material, figure S7), is located in close proximity to the triad Y-D-R, so the change from Leu to Pro may alter the interactions of the triad or the surface epitope. However to confirm this analysis, a similar study as the one conducted by Li *et al.* would need to be repeated for these particular mutations, and the occurrence of ER trapping investigated [19]. The mutation A876S has the potential to destabilize one binding site (Cleft 1), with potential steric clashes of the Ser-Oγ atom group with the β-Ionone ring of the retinoid ligand (approx. 3.0 Å, instead of the 3.8 Å of the Cβ of the Alanine residue).

### 4.2. Docking—pRBP3 ligand binding

The major limitation of our 3.67 Å reconstruction of pRBP3 is that the endogenous ligands cannot be identified. Thus, the binding sites, and ligand-binding network, of RBP3 remain elusive. Nevertheless, our work permits speculation on the influence of the putative-binding sites on the module-to-module communication. The two sites previously predicted at the hydrophobic cavity within domain B and in the hydrophobic groove between domains A and B [26,28,29] ranked high in our screening, using 11c-RAL, at-ROL, DHA and oleic acid (figure 6). For fatty acids, there is currently a co-crystal structure (PDB id: 4LUR) that indicates that the three helixes of domain A could accommodate a molecule of oleic acid [26], but our docking trials did not identify that site (figure 4*d*). Our docking screen revealed not only the expected binding site at a hydrophobic cavity within domain B of RBP3 (figure 6*a*,*b*), which bears similarities to the ligand site of cellular retinaldehyde-binding protein CRALBP (PDB id: 3HY5 [54]) but also many additional clefts. Our analysis showed strong energy barriers between cavities and clefts (a greater absolute value; i.e. smaller score value corresponds to a stronger binding force) but a gradient of energy values within the clefts (electronic supplementary material, table S1). The clefts that are located at domain interfaces are particularly relevant for this work, as they may induce conformational changes upon ligand binding. The average binding energy (across 20 binding modes) are −6.13, −5.98, −5.34 and −4.40 kcal mol^−1^ for 11c-RAL, at-ROL, DHA and OA, respectively.

Considering the clefts that position ligands at interfaces between domains, we highlight those between domains A and B of Module 3 (Clefts 1 and 2; figure 6*c*,*d*), which may undergo conformational changes to better accommodate the ligands. In both of these clefts, stronger average binding energies, on the order of −2.15 kcal mol^−1^, are calculated for 11c-RAL and at-ROL than for DHA or oleic acid, which may help explain the different behaviour of pRBP3 towards the ligands and also the different conformational outcomes detected on our SAXS analysis. Similar situations are observed for Cleft 2, with an energy difference on the order of −1.45 kcal mol^−1^; Cleft 3 (at the interface of domain B of Module 3 and domain A of Module 4), with an energy difference on the order of −1.5 kcal mol^−1^; and Cleft 7 (at the interface of domain B of Module 2 and domain A of Module 3), with an energy difference on the order of −1.3 kcal mol^−1^.

Of note, our structure indicates that the clefts between modules are more stable, supported by a bent but rigid and conformationally conserved long helix that connects them (figure 4), whereas the clefts between domains within single modules likely are the more flexible hinges in the full-length protein (figures 2*c* and 5*a*), with the linking loops being interspersed with multiple dyads of glycines (figure 4*c*). Moreover, single modules that appear to have shallower grooves between domains are perhaps more prone to having DHA-displacing retinoids, as reported previously [55,56]. These results also help explain the different number of binding sites that have been reported over the years [47,55,57–64].

### 4.3. Ligand-induced conformational changes

Our in-solution SAXS measurements indicate that in addition to the intrinsically flexible termini observed on the pRBP3 cryoEM structure, the loading of retinoids induces some conformational change in the protein. However, the change, according to SAXS, is smaller than observed by negative staining EM or suggested by sedimentation analysis [44], where a 24 nm length was reported for the extended conformation, compared with our measured *D*_max_ of <20 nm. The negative staining results, which we replicated with our protein preps (not shown), can be quickly rejected because of the dehydration imposed on the proteins by that procedure. The sedimentation studies, however, were performed *via* sucrose-gradient centrifugation. The sucrose should protect proteins from dehydration if used at appropriate concentrations; perhaps in other studies, it was used in a range that induced protein unfolding due to osmotic pressure.

The current SAXS experiments were the first to study the solution behaviour of pRBP3 alone and in complex with retinoids and fatty acids. We found that upon binding with 11c-RAL (8 μM) and at-ROL (12 μM), pRBP3 exhibits conformational dynamics in solution and can exist both as an opened form (11c-RAL; figure 7*j*), and as a partially opened form (at-ROL; figure 7*k*). We surmise that lower concentrations (e.g. 2, 4 and 6 μM) of retinoids bind only to the junction sites of Modules 1−2 and Modules 3−4, as these sites are easily accessible and least likely to require conformational changes to accommodate binding. Higher concentrations (8 and 12 μM) of retinoids bind to all available junction sites and would be responsible for the conformational extension of the protein, as more retinoids would be available to bind.

## 5. Conclusion

To our knowledge, this study is the first to investigate the properties and structure of porcine RBP3. In addition to determining the cryoEM structure of the full-length native RBP3 protein, we confirm its glycosylation and characterize the relationship between its binding of retinoids and fatty acids. In all measured parameters, the porcine variant mimics the more completely characterized bovine variant. The capacity of RBP3 to load different retinoids and fatty acids, the ability of the latter to displace the former and the conformational changes dependent on ligand identity might be the basis for the loading and unloading of retinoids (and potentially DHA) to the intended cell types bordering the IPM. Thus, RBP3 complexes merit further investigation.

## Data Availability

The coordinates and cryoEM data have been deposited to the PDB and EMDB databases, with the following identifier: 9FRX and EMD−50552, respectively (for details, see table 1). Supplementary material is available online [65].
